# Evaluation of surveillance case definition in the diagnosis of leptospirosis, using the Microscopic Agglutination Test: a validation study

**DOI:** 10.1186/1471-2334-9-48

**Published:** 2009-04-22

**Authors:** Dinesh LB Dassanayake, Harith Wimalaratna, Suneth B Agampodi, Veranja C Liyanapathirana, Thibbotumunuwe ACL Piyarathna, Bimba L Goonapienuwala

**Affiliations:** 1 Teaching Hospital Kandy, Kandy, Sri Lanka; 2 Department of Community Medicine, Faculty of Medicine, University of Peradeniya, Peradeniya, Sri Lanka

## Abstract

**Background:**

Leptospirosis is endemic in both urban and rural areas of Sri Lanka and there had been many out breaks in the recent past. This study was aimed at validating the leptospirosis surveillance case definition, using the Microscopic Agglutination Test (MAT).

**Methods:**

The study population consisted of patients with undiagnosed acute febrile illness who were admitted to the medical wards of the Teaching Hospital Kandy, from 1^st ^July 2007 to 31^st ^July 2008. The subjects were screened to diagnose leptospirosis according to the leptospirosis case definition. MAT was performed on blood samples taken from each patient on the 7^th ^day of fever. Leptospirosis case definition was evaluated in regard to sensitivity, specificity and predictive values, using a MAT titre ≥ 1:800 for confirming leptospirosis.

**Results:**

A total of 123 patients were initially recruited of which 73 had clinical features compatible with the surveillance case definition. Out of the 73 only 57 had a positive MAT result (true positives) leaving 16 as false positives. Out of the 50 who didn't have clinical features compatible with the case definition 45 had a negative MAT as well (true negatives), therefore 5 were false negatives. Total number of MAT positives was 62 out of 123. According to these results the test sensitivity was 91.94%, specificity 73.77%, positive predictive value and negative predictive values were 78.08% and 90% respectively. Diagnostic accuracy of the test was 82.93%.

**Conclusion:**

This study confirms that the surveillance case definition has a very high sensitivity and negative predictive value with an average specificity in diagnosing leptospirosis, based on a MAT titre of ≥ 1: 800.

## Background

Leptospirosis is endemic in both urban and rural areas of Sri Lanka and there had been many out breaks in the recent past. Two thousand one hundred and eighty seven cases were reported in 2007 in Sri Lanka while 147 cases were reported from the Kandy District[1].

Confirmation of leptospirosis in a patient requires serological tests, which become positive usually after 7 days of the illness [2]. Direct microscopic visualization of blood is of value only during the first few days of the acute illness, when leptospiremia occurs.

Dark-field microscopic examination of body fluids such as blood, urine, CSF, and dialysate fluid has been used, but is both insensitive and lacks specificity. The drawbacks of dark-field microscopy on clinical specimens as a diagnostic tool have been that both false positive and false negative diagnosis can be easily made even in experienced hands[3]. Therefore it is unhelpful in the management of patients, as the treatment should begin as early as possible preferably within 5 days to prevent complications[4]. If the patient is not treated for the severe form within 2–3 days after the onset of illness, it may progress in severity and sometimes be fatal. The beginning of early pertinent antimicrobial therapy within 4–5 days after the onset of illness, proper supportive therapy and use of dialysis to treat renal failure has reduced the leptospirosis-related mortality[4]. Therefore clinical criteria have to be employed to screen patients and to decide on treatment.

Medical professionals, especially primary care physicians, who are primarily responsible for the diagnosis and treatment, need to know about the early symptoms and signs, and early clinical laboratory findings.

Validation of clinical criteria using microscopic agglutination test as the gold standard has been done in 1995 by Brato and colleagues in the Philippine General Hospital [5], but such a study has not been conducted in Sri Lanka. This study was aimed at validating the leptospirosis surveillance case definition, using a Microscopic Agglutination Test (MAT) titre of ≥ 1:800 to confirm leptospirosis.

## Methods

Leptospirosis case definition is based on the diagnosis criteria formulated in International Classification of Diseases (ICD)10 (A 27)[6]. In this study case definition was evaluated with regard to sensitivity, specificity, and predictive values in diagnosing leptospirosis using MAT which is the only available method of confirming the disease in the government sector. Reported sensitivity and specificity of the MAT are as high as 92% and 95%, respectively. Positive predictive values of 95% and negative predictive values of 100% also have been documented[7]

### Study Setting

This study was carried out in wards 33 and 35 (Medical) of the Teaching Hospital Kandy, which is a tertiary care hospital. This medical unit treats about 10000 patients per year and 10% of the admissions are due to fever. Ethical approval was granted by the Ethical Committee of the Teaching Hospital Kandy. {No: AB (II)/ETHICAL/2007}

Patients with undiagnosed acute febrile illness who were admitted to wards 33 and 35, from 1^st ^July 2007 to 31^st ^July 2008 were the study population. All patients presented with an acute febrile illness, with fever less than 15 days were included in the sample and patients with a definitive diagnosis as a cause for the acute febrile illness were excluded. Patients were screened for eligibility criteria by the first author. Data collection was done prospectively for a period of one year. An interviewer administered questionnaire, symptom check list and data extraction form was used for the collection of data.

### Procedure

After the initial assessment for eligibility criteria the cases were screened using a symptom check list and interviewer administered questionnaire to diagnose leptospirosis according to leptospirosis case definition. Surveillance case definition for diagnosis of leptospirosis which was published by the Epidemiology Unit was used for the present study[6].

Acute febrile illness, with headache, myalgia, and prostration associated with any of the following.

1. Conjunctival suffusion/haemorrhage

2. Meningial irritation

3. Anuria/Oliguria/haematuria/proteinuria

4. Hemorrhage – intestinal bleeding, lung bleeding or purpuric rash

5. Cardiac arrhythmias/failure

Plus:

History of exposure to infected animals/environment contaminated with animal urine was considered as leptospirosis according to the case definition.

A blood sample was taken from each patient irrespective of the working diagnosis on the 7^th ^day of fever. Informed written consent was obtained from patients before blood sampling. Blood samples were sent to the Medical Research Institute (MRI) in Colombo for MAT. Leptospirosis was confirmed using the genus specific MAT. The MRI uses the non pathogenic Patoc strain of *Leptospira biflexa *for MAT. A positive MAT test was defined as a titre of ≥1: 800 in acute phase serum. This is the titre specified by the MRI which is the national reference laboratory[8].

Data was entered in to an Excel data base and analyzed in Epi info and Open Epi. Descriptive analysis was done using percentages and validity of the screening test was assessed using sensitivity, specificity, predictive values, and accuracy.

## Results

A total of 123 patients were initially recruited of which 73 had clinical features compatible with surveillance case definition. Out of the 73 only 57 had positive MAT result (true positives) leaving 16 as false positives. Out of the 50 who didn't have clinical features compatible with case definition 45 had a negative MAT as well (true negatives), therefore 5 were false negatives. Total number of MAT positives (confirmed cases) was 62 out of 123.

### Characteristics of the Confirmed Cases

Mean age of the confirmed cases of leptospirosis was 39 years with a standard deviation of 17.9. The male to female ratio was 3:1. Table 1 shows age and sex distribution of the leptospirosis patients.

**Table 1:** Age and sex distribution of confirmed cases

	N	%
**Sex**		

Male	47	75.8

Female	15	24.2

**Age**		

<20	10	16.1

20 – 39	21	33.9

40 – 59	27	43.5

>60	3	4.8

**Figure 1:**
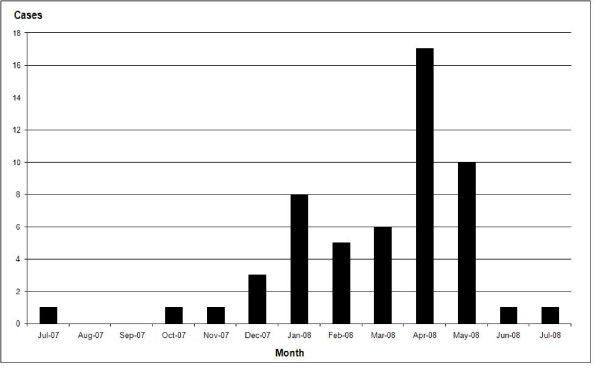
**Incidence of patients according to month**.

Majority of cases were diagnosed during the period of November to May. The highest number of cases was reported in the month of April, which amounted to17 cases. Figure 1 illustrates the distribution of cases over the study period.

Signs and symptoms of MAT positive cases are presented in Table 2.

**Table 2:** Prevalence of clinical symptoms and signs among confirmed cases

Symptoms and signs	No	%
Fever	62	100

Myalgia	60	97

Headache	57	90

Conjunctival suffusion	40	65

Systolic blood pressure <100 mm Hg	25	40

Oliguria	17	27

Icterus	12	19

Hepatomegally	10	16

Chest pain	6	10

Bleeding	5	8

Shortness of breath	4	6

Haematuria	3	5

Rash	2	3

Palpitations	1	2

Conjunctival haemorrhage	1	2

Arrhythmia	1	2

Liver flaps	1	2

Table 3 shows the distribution of the patients, according to the positivity of the clinical criteria of surveillance case definition, and MAT.

**Table 3:** 4 × 4 table showing number of patients meeting clinical criteria of case definition and MAT results

	MAT	MAT	Total
	positive	negative	
**Surveillance Case**			
**Definition positive**	57	16	**73**

**Surveillance Case**			
**Definition negative**	5	45	**50**

**Total**	**62**	**61**	**123**

Sensitivity and specificity of the clinical criteria of surveillance case definition was 92% and74% respectively. Table 4 shows the parameter estimates of the case definition with 95% confidence Intervals (CI).

**Table 4:** Parameter estimates of leptospirosis surveillance case definition

Parameter	Estimate	Lower – Upper 95% confidence intervals
**Sensitivity**	91.94%	82.47, 96.51

**Specificity**	73.77%	61.56, 83.16

**Positive Predictive Value**	78.08%	67.32, 86.03

**Negative Predictive Value**	90%	78.64, 95.65

**Diagnostic Accuracy**	82.93%	75.31, 88.55

## Discussion

Main aim of the study was to validate the case definition. The results show a sensitivity of 92% which is a high value. But the specificity was 73.7%. Ninety five percent confidence interval for sensitivity was between 82 and 96. High sensitivity has also given the high negative predictive value of 90%. This finding contradict the findings of the study by Brato et al which showed a sensitivity of only 33% (95% CI, 19–51); specificity 65% (95% CI, 39–85%) [5]. Clinical criteria used in our study were slightly different to the criteria used by Brato et al. In addition they used a scoring system to decide on the positivity and negativity of clinical criteria [5]. These two factors would have contributed to the discrepancy of the results.

These results confirm that the case definition has a very high sensitivity and negative predictive value in diagnosing leptospirosis. This is a valuable finding for a resource poor setting like ours in Sri Lanka, as most of the diagnostic and therapeutic decisions are based on clinical findings rather than investigations. It is also important as the confirmatory test MAT takes at least 7 days to be positive[2], and the complications are higher if the treatment is delayed more than 5 days[4]. Therefore crucial decision on treatment of patients are based on clinical criteria rather than serological studies.

The study has also confirmed the male predominance which has been described in early studies[5]. This is because males are exposed more to agrarian occupations. Age distribution shows that the working age group is affected more. This signifies the impact of disease on economy.

Time distribution is peculiar in that the most number of cases were reported in the months of January, April and May. This correlates with the agrarian activities that occur with rain. The surge of patients in January could be due to the exposure of patients while sowing of paddy fields during the eastern monsoon in November and December. Increased numbers in April and May could be due to the exposure during harvesting as well as the sowing with the beginning of inter-monsoon rains. As August and September have a dry weather no farming is done hence the exposure is minimum. This would have been the reason not to have patients in those months.

Leptospirosis case definition is based on ICD 10 criteria put forward by the World Health Organization [6]. All patients included in the study had fever. The commonest symptoms of those who were confirmed as having leptospirosis were, fever, mayalgia, prostration and headache. Substantial proportion had oliguria. Other symptoms were not that common. The commonest sign was conjunctival suffusion, while many had hypotension (systolic blood pressure <100 mmHg), icterus and hepatomegally. In previous studies in China, Barbados and Brazil, jaundice was much more common than that found among our patients[9]. The same studies showed a significant proportion had pulmonary hemorrhage a clinical feature which was not found in our study population [9]. Presence of four major symptoms and one out of five minor clinical features with exposure to leptospirosis have shown to be a sensitive index for screening patients.

## Limitations of the study

Due to the limited study period sample size was not calculated, and only 123 patients were included in the study which is a small sample. But the confidence intervals were not that wide, therefore sample size is adequate for the interpretation of results.

Three patients suspected of having leptospirosis died before serology could be performed, and were not included in the study, which would have caused some bias in the sample.

Ideally, the gold standard should be the isolation of the leptospira in culture [3]. This was not used in this study because unavailability and the culture yield was very low[3]. Pre admission antibiotic use which is common in Sri Lanka would result in negative cultures and further rendering it an unreliable test. MAT was the only laboratory test that was available to us for the confirmation of leptospirosis, which is also the test specified by the epidemiology unit [6].

Four fold rise in paired sera should be demonstrated to confirm the disease [6,9]. It was difficult to get down the patients for a second convalescent sample as they didn't return for follow up once discharged. We had to be confined to use the acute phase sera only. This would have caused some reduction in sensitivity and specificity of this test. Literature supports the use of single acute phase titre of ≥ 1:800 to diagnose leptospirosis [9,10]. This is the MAT titre specified by the MRI to diagnose leptospirosis if acute phase serum is used[8].

MAT used in our reference laboratory is genus specific, and is not serovar specific. Therefore serogroups could not be typed. The Same method had been used by Brato and collegues in 1995 for his study but with a different titre [5]. However the popular belief that different serovars produced different syndromes has been refuted[9].

This study confirms the higher sensitivity of surveillance case definition in the diagnosis of leptospirosis. Sensitivity can be further increased by adding simple laboratory criteria like microscopic haematuria, and thrombocytopaenia. Further validation studies with bigger samples and utilization of four fold rise in MAT titre for confirmation would clarify most of the problems encountered during our study.

## Conclusion

This study confirms that the surveillance case definition has a very high sensitivity, and negative predictive value with an average specificity in diagnosing leptospirosis, based on a MAT titre of ≥ 1: 800.

## Competing interests

The authors declare that they have no competing interests.

## Authors' contributions

DLBD was involved in designing, acquisition of data and drafting the manuscript of the study. HW conceived the study, reviewed the manuscript and approved the final manuscript. SBA was involved in statistical analysis, interpretation of data and review of the manuscript. VCL was involved in the acquisition of data, and designing the study. TACLP was engaged in designing the study, and interpretation of data. BLG was involved in data acquisition, and review of the manuscript. All authors approved the final manuscript.

## Author information

DLBD: Is the corresponding author and is a registrar in Medicine attached to wards 33 and 35 of the Teaching Hospital Kandy. He has an MBBS degree and is enrolled in the MD Medicine Training Program in the Postgraduate Institute of Medicine, Colombo.

HW is the Consultant Physician in charge of the Medical Unit, wards 33 and 35 in the Teaching Hospital Kandy. He has obtained MD, FRCP and FCCP and is a member of the Collage of Physicians Sri Lanka.

SBA has an MBBS and an MSc in Community Medicine. He is currently a doctoral trainee in Community Medicine, attached to Department of Community Medicine, Faculty of Medicine, University of Peradeniya.

VCL is an MBBS graduate working as an Intern House Officer in the Teaching Hospital Kandy. She is also following an MPhil in the Department of Microbiology, Faculty of Medicine, University of Peradeniya.

TACLP is an MBBS graduate working as an Intern House Officer in the Teaching Hospital Kandy.

BLG is an MBBS graduate working as an Intern House Officer in the Teaching Hospital Kandy.

## Pre-publication history

The pre-publication history for this paper can be accessed here:

http://www.biomedcentral.com/1471-2334/9/48/prepub
